# Wide but Variable Distribution of a Hypervirulent Campylobacter jejuni Clone in Beef and Dairy Cattle in the United States

**DOI:** 10.1128/AEM.01425-17

**Published:** 2017-12-01

**Authors:** Yizhi Tang, Richard J. Meinersmann, Orhan Sahin, Zuowei Wu, Lei Dai, James Carlson, Jodie Plumblee Lawrence, Linda Genzlinger, Jeffrey T. LeJeune, Qijing Zhang

**Affiliations:** a Departments of Veterinary Microbiology and Preventive Medicine, Iowa State University, Ames, Iowa, USA; b Russell Research Center, USDA Agricultural Research Service, BEAR-RU, Athens, Georgia, USA; c Department of Veterinary Diagnostic and Production Animal Medicine, Iowa State University, Ames, Iowa, USA; d National Wildlife Research Center, USDA APHIS, Fort Collins, Colorado, USA; e Food Animal Health Research Program, The Ohio State University, Wooster, Ohio, USA; The Pennsylvania State University

**Keywords:** Campylobacter jejuni, cattle, clone SA, prevalence

## Abstract

Campylobacter jejuni clone SA is the major cause of sheep abortion and contributes significantly to foodborne illnesses in the United States. Clone SA is hypervirulent because of its distinct ability to produce systemic infection and its predominant role in clinical sheep abortion. Despite the importance of clone SA, little is known about its distribution and epidemiological features in cattle. Here we describe a prospective study on C. jejuni clone SA prevalence in 35 feedlots in 5 different states in the United States and a retrospective analysis of clone SA in C. jejuni isolates collected by National Animal Health Monitoring System (NAHMS) dairy studies in 2002, 2007, and 2014. In feedlot cattle feces, the overall prevalence of Campylobacter organisms was 72.2%, 82.1% of which were C. jejuni. Clone SA accounted for 5.8% of the total C. jejuni isolates, but its prevalence varied by feedlot and state. Interestingly, starlings on the feedlots harbored C. jejuni in feces, including clone SA, suggesting that these birds may play a role in the transmission of Campylobacter. In dairy cattle, the overall prevalence of clone SA was 7.2%, but a significant decrease in the prevalence was observed from 2002 to 2014. Whole-genome sequence analysis of the dairy clone SA isolates revealed that it was genetically stable over the years and most of the isolates carried the tetracycline resistance gene *tet*(O) in the chromosome. These findings indicate that clone SA is widely distributed in both beef and dairy cattle and provide new insights into the molecular epidemiology of clone SA in ruminants.

**IMPORTANCE** C. jejuni clone SA is a major cause of small-ruminant abortion and an emerging threat to food safety because of its association with foodborne outbreaks. Cattle appear to serve as a major reservoir for this pathogenic organism, but there is a major gap in our knowledge about the epidemiology of clone SA in beef and dairy cattle. By taking advantage of surveillance studies conducted on a national scale, we found a wide but variable distribution of clone SA in feedlot cattle and dairy cows in the United States. Additionally, the work revealed important genomic features of clone SA isolates from cattle. These findings provide critically needed information for the development of preharvest interventions to control the transmission of this zoonotic pathogen. Control of C. jejuni clone SA will benefit both animal health and public health, as it is a zoonotic pathogen causing disease in both ruminants and humans.

## INTRODUCTION

Campylobacter jejuni is a major zoonotic bacterial pathogen and primarily causes foodborne enteritis in humans (1, 2). The organism is widely distributed across a broad range of animal species, including livestock, poultry, and wildlife, and is transmitted to humans mainly via consumption of contaminated food, water, and milk (2). As reported by the Centers for Disease Control and Prevention's (CDC) FoodNet surveillance program in 2016, Campylobacter ranked second (12.97 per 100,000 population) among the causes of laboratory-confirmed bacterial foodborne illnesses in the United States (3). Poultry, especially market age broiler chickens, are frequently colonized by C. jejuni, resulting in carcass contamination in processing plants (4, 5). Consequently, poultry meat is considered a major source of infection for human campylobacteriosis.

In addition to poultry, cattle also serve as an important reservoir for Campylobacter. Bovine Campylobacter contributes significantly to both outbreak and sporadic cases of campylobacteriosis in humans (6, 7). Campylobacter can be transmitted from cattle to humans via multiple routes, including direct contact (e.g., petting zoo and occupational exposure), consumption of unpasteurized milk (and associated dairy products), and environmental contamination (water, produce, etc.) (8 – 10). Molecular typing of C. jejuni isolates using multilocus sequence typing (MLST) attributed approximately 40% of sporadic human cases to cattle sources in the United Kingdom (11). The contribution of bovine Campylobacter to outbreaks of human campylobacteriosis is even more prominent because Campylobacter from cattle feces frequently contaminates raw milk (9, 12 – 14). Ruminant Campylobacter may also contaminate water supplies via agricultural runoff, leading to large waterborne outbreaks (8). Of note, red meat is infrequently contaminated by Campylobacter (15) and does not appear to play a major role in the transmission of Campylobacter to humans. Additionally, ruminants are an integral part of Campylobacter ecology and may serve as a source of Campylobacter transmission to the environment and other farm animals, such as poultry. Thus, poultry and cattle are the two most important animal reservoirs for this zoonotic pathogen.

Campylobacter is highly prevalent in both beef and dairy cattle in the United States and worldwide (10, 16 – 20). In cattle, Campylobacter is mainly carried in the intestinal tract and less frequently can be isolated from the rumen, gallbladder, and bile (11, 21). The predominant Campylobacter species isolated from cattle is C. jejuni, followed by C. coli (10, 22 – 25). Isolation rates vary with country, herd size and type, age of animals, season, and confinement level (10, 25). In the United States, several nationwide surveillance studies of cattle (National Animal Health Monitoring System [NAHMS] Dairy 1996, 2002, and 2007 and Feedlot'99) indicated that fecal carriage rates ranged from 15 to 50% and the majority of the tested operations (herds/farms/feedlots) were positive for Campylobacter (17, 24, 26). Several other studies conducted in different states in the United States also revealed a similar range of prevalence (between 20 and 60% at the fecal sample level) of Campylobacter in feedlot cattle and dairy cattle (18, 22, 23, 25, 27).

Although Campylobacter mainly colonizes in the gastrointestinal (GI) tract in animals, it may translocate across the intestinal epithelial barrier, leading to systemic infection, such as bacteremia and abortion in small ruminants and occasionally in humans (28). Indeed, Campylobacter infection is one of the most prevalent causes of ovine abortion in the United States and worldwide, with an overall abortion rate of 5% to 50% (average, 23.2%) in affected flocks (29). Historically, Campylobacter fetus subsp. fetus was the major cause of Campylobacter-associated ovine abortion. However, studies conducted during late 1980s and early 1990s in the United States revealed a progressive increase in isolation of C. jejuni from aborted sheep placentas (30, 31). Recently, our studies demonstrated that a single hypervirulent tetracycline-resistant C. jejuni clone (named clone SA) has emerged as the predominant cause of Campylobacter-associated ovine abortions and is responsible for >90% of the clinical abortion cases in the United States (29, 32, 33). The hypervirulence of clone SA is related to its ability to translocate across the intestinal epithelium, producing systemic infection and clinical abortion (33). Additionally, clone SA was also associated with bovine and goat abortion cases in the United States (29, 34). Importantly, C. jejuni clone SA has been implicated in a number of cases of foodborne illnesses, both outbreaks and sporadic cases, in the United States (34). These findings clearly indicated that C. jejuni clone SA is an important pathogen for both animal health and food safety in the United States and suggest that cattle may serve as a major reservoir for its zoonotic transmission.

Despite the obvious significance of C. jejuni clone SA to ruminant health and food safety, little information is available about its distribution in beef and dairy cattle, which represents an important knowledge gap in our understanding of the overall epidemiology and this particular zoonotic risk. To close this knowledge gap and facilitate the control of C. jejuni clone SA, we conducted a before-after controlled impact (BACI) study, with repeated sampling of 35 feedlots located in various geographical regions on two different occasions. Additionally, we analyzed the Campylobacter isolates in the collections of the NAHMS 2002, 2007, and 2014 dairy studies (17, 35). The purposes of this work were to (i) investigate the overall prevalence of Campylobacter in feedlot cattle and evaluate the effect of starling control intervention on the occurrence and spread of Campylobacter in feedlot operations and (ii) determine the occurrence and distribution of C. jejuni clone SA in feedlot and dairy cattle.

## RESULTS

### Overall prevalence of Campylobacter in feedlot cattle.

In total, 2,298 (72.1%) out of 3,184 fecal samples from feedlots were positive for Campylobacter. The overall prevalence rates of Campylobacter were 69.2% (554/800), 71.9% (414/576), 70.0% (210/300), 78.2% (593/758), and 70.3% (527/750) in Iowa, Texas, Missouri, Colorado, and Kansas, respectively. The Campylobacter prevalence rates among the states were not statistically different (*P* > 0.05). Of the 2,298 Campylobacter isolates, 1,886 (82.1%) were determined to be C. jejuni by PCR. In each of the states, 487 (87.9%), 367 (88.6%), 191 (91.0%), 438 (73.9%), and 403 (76.5%) were identified as C. jejuni, respectively (Table 1), indicating that C. jejuni was the predominant Campylobacter species isolated from cattle feces.

**TABLE 1:** Prevalence of Campylobacter jejuni and C. jejuni clone SA in feces of feedlot cattle and starlings in the United States

State	% prevalence of Campylobacter in cattle (no. of isolates/total no. of samples)	No. (%) of cattle isolates		% prevalence of Campylobacter in birds (no. of isolates/total no. of samples)	No. (%) of bird isolates	
		C. jejuni^*a*^	Clone SA^*b*^		C. jejuni^*a*^	Clone SA^*b*^
Iowa	69.2 (554/800)	487 (87.9)	16 (3.3)	NA^*c*^	NA	NA
Texas	71.9 (414/576)	367 (88.6)	42 (11.4)	25.8 (23/89)	23 (100)	1 (4.3)
Missouri	70.0 (210/300)	191 (91.0)	15 (7.9)	90.9 (10/11)	10 (100)	0
Colorado	78.2 (593/758)	438 (73.9)	14 (3.2)	55.0 (11/20)	10 (91.0)	0
Kansas	70.3 (527/750)	403 (76.5)	23 (5.7)	23.3 (7/30)	7 (100)	0
Total	72.2 (2,298/3,184)	1,886 (82.1)	110 (5.8)	34.0 (51/150)	50 (98.0)	1 (2.0)

a The percentage is the proportion of C. jejuni isolates among the Campylobacter isolates.

b The percentage is the proportion of clone SA isolates among the C. jejuni isolates.

c NA, starling samples were not available.

### Effect of starling intervention on Campylobacter prevalence in feedlot cattle.

Delineation of the prevalence data pre- and post-starling control intervention is shown in Table 2. The intervention program did not appear to significantly affect the overall prevalences of Campylobacter (*P* = 0.10) and C. jejuni (*P* = 0.29) in the feedlot cattle. Before intervention, the overall prevalence of Campylobacter (69.1% [1,044/1,510 samples tested]) and the relative prevalence of C. jejuni (79.3% [828/1,044]) were comparable to those observed postintervention (74.9% and 84.4%, respectively). Also, analysis of data by each state separately indicated no significant differences pre- and postintervention in the prevalence rates for overall Campylobacter and C. jejuni (Table 2).

**TABLE 2:** Prevalence of Campylobacter isolated from fecal samples of feedlot cattle before and after starling intervention

State	Preintervention				Postintervention			
	No. of samples tested	No. Campylobacter positive (%)	No. of C. jejuni isolates (%)^a^	No. of clone SA isolates (%)^b^	No. of samples tested	No. Campylobacter positive (%)	No. of C. jejuni isolates (%)^a^	No. of clone SA isolates (%)^b^
Iowa	400	280 (70.0)	245 (87.5)	7 (2.9)	400	273 (68.3)	242 (88.6)	9 (3.7)
Texas	250	170 (68.0)	144 (84.7)	12 (8.3)	326	244 (74.8)	223 (91.4)	30 (13.5)
Missouri	150	85 (56.7)	72 (84.7)	5 (6.9)	150	125 (83.3)	119 (95.2)	10 (8.4)
Colorado	360	274 (76.1)	202 (80.3)	6 (3.0)	398	319 (80.2)	236 (74.0)	8 (3.4)
Kansas	350	235 (67.1)	165 (63.8)	6 (3.6)	400	292 (73.0)	238 (81.5)	17 (7.1)
Total	1,510	1,044 (69.1)	828 (79.3)	36 (4.3)	1,674	1,253 (74.9)	1,058 (84.4)	74 (7.0)

a The percentage is the proportion of C. jejuni isolates among the Campylobacter isolates.

b The percentage is the proportion of clone SA isolates among the C. jejuni isolates.

### Prevalence of C. jejuni clone SA in feedlot cattle.

**FIG 1:**
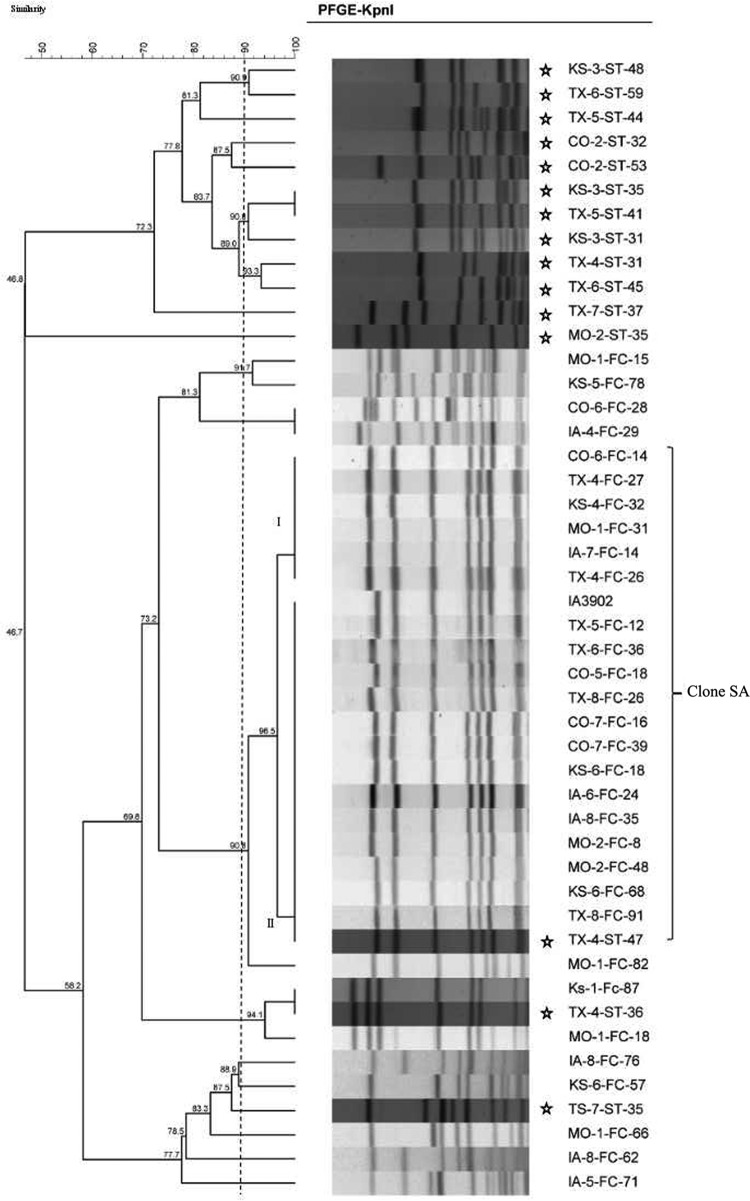
Dendrogram showing the PFGE patterns (KpnI) of C. jejuni isolates from feces of feedlot cattle and starlings. The clone SA strains are represented by two closely associated PFGE patterns (I and II), as was the case for sheep clone SA isolates (29). IA3902 is a known isolate of clone SA and was used as a reference. The isolates' names are listed on the right of the dendrogram. Starling isolates are marked with stars. TX, Texas; CO, Colorado; MO, Missouri; IA, Iowa; KS, Kansas; FC, feedlot cattle; ST, starling. The numbers in the names of the isolates are arbitrary numbers assigned to feedlots and samples. Please note that the starling isolates represent the total C. jejuni population isolated from the birds, while the cattle isolates included in the PFGE analysis were preselected for putative clones SA by the PCR.

Initial screening of the C. jejuni isolates for putative clone SA using PCR revealed that 8.7% (164/1,886) of the isolates were positive by PCR. As this PCR is not 100% specific for clone SA, pulsed-field gel electrophoresis (PFGE) was performed to confirm their identity as clone SA. Of the 164 isolates initially identified by the PCR as putative clone SA, 110 (67.1%) had patterns that matched to the known subtypes of clone SA—I and II (Fig. 1)—which is in accordance with our previously published results (29). Additionally, MLST was performed on a subset of these isolates in both PFGE subtypes, which identified all of them as sequence type (genotype) 8 (ST-8), which confirmed the PFGE typing result. MLST was also performed on seven isolates with non-clone SA PFGE patterns, two of which with one band difference from clone SA were also identified as ST-8 genotype, while the remaining five isolates were identified with sequence types different from ST-8, including ST-2876, ST-93, ST-239, and ST-14.

Based on the genotyping results, a relative prevalence of 5.8% (110 out of 1,886 C. jejuni isolates) and an absolute prevalence of 3.5% (110 out of 3,184 total samples tested) were calculated for clone SA occurrence in the feedlot cattle surveyed in this study. The absolute prevalence rates of clone SA varied by state: 1.8% (14/758) in Colorado, 2.0% (16/800) in Iowa, 3.1% (23/750) in Kansas, 5.0% (15/300) in Missouri, and 7.3% (42/576) in Texas (Table 1). The chi-square test revealed that at least one state is significantly different from the rest (*P* < 0.0001). The relative prevalence rates of clone SA also varied by state: Iowa, 2.9% (16/554); Texas, 10.1% (42/414); Missouri, 7.1% (15/210); Colorado, 2.4% (14/593); and Kansas, 4.4% (23/527) (*P* < 0.0001). However, starling intervention did not affect the prevalence of clone SA on the surveyed farms (Table 2). Clone SA strains were isolated from at least half of the feedlots surveyed in each state, in the range of 1 to 28 isolates per feedlot (result not shown). Although PFGE was performed only on putative clone SA isolates identified by PCR, the non-clone SA C. jejuni isolates showed diverse PFGE patterns (Fig. 1), suggesting the overall genetic diversity of C. jejuni isolates from feedlot cattle. These findings indicate that C. jejuni clone SA is widely distributed and constitutes a substantial portion (∼6%) of the total C. jejuni population in feedlot cattle.

### Presence of Campylobacter, including clone SA, in starlings.

European starlings are commonly found on farms, serving as a potential transmission vehicle for Campylobacter (36). To investigate whether they may be a source of farm cattle infection of C. jejuni clone SA, fecal samples from European starlings present on 7 feedlots were tested for Campylobacter occurrence. Of note, the same feedlots were also sampled for cattle feces at or about the time of the starling survey. Of the 150 total starling fecal samples tested, 51 (34%) were positive for Campylobacter; 50 (98%) isolates were identified as C. jejuni by PCR, and the remaining isolate was of a species other than C. jejuni or C. coli. Initial screening using PCR identified 1 of the 50 C. jejuni isolates to be a putative clone SA (Table 1). This isolate and an additional 14 randomly chosen C. jejuni isolates were analyzed by PFGE, which confirmed that the putative clone SA isolate identified by PCR had a PFGE pattern indistinguishable from IA3902 of clone SA (Fig. 1). MLST analysis further identified this starling isolate as ST-8, indicating that it was a clone SA isolate. All together, these results indicate that starlings carry diverse C. jejuni strains and can serve as a vector for transmission of Campylobacter, including clone SA, within and between farms.

### Prevalence of clone SA in dairy cattle.

A previous study reported that raw milk was the main source of foodborne illness outbreaks caused by C. jejuni clone SA (34), suggesting the presence of clone SA in dairy cattle. Thus, we performed an analysis of the retrospective collections of Campylobacter isolates derived from dairy cattle by NAHMS. In 2002, 2007, and 2014, the NAHMS conducted national surveillance studies on Campylobacter prevalence in dairy cattle (17, 35). In total, 205, 627, and 576 C. jejuni isolates collected in 2002, 2007, and 2014, respectively, were available for clone SA screening. Of these C. jejuni collections, 11.2% (23/205), 10.5% (66/627), and 6.8% (39/576) of isolates were initially identified as putative clone SA by PCR (*n* = 128 total), respectively. All but three (one from the Dairy 2007 study and two from the Dairy 2014 study) of the putative clone SA isolates were subjected to whole-genome sequencing (WGS) analysis. Overall, 16 STs were identified among the genome-sequenced isolates (Table S1). Of the 125 isolates sequenced, 102 (81.6%) were confirmed as clone SA, which gave a relative prevalence of 7.2% (102/1,408) for clone SA among the C. jejuni isolates from U.S. dairy cattle. These clone SA isolates included 21 (10.2%) from the Dairy 2002 study, 55 (8.8%) from the Dairy 2007 study, and 26 (3.2%) from the Dairy 2014 study (Table 3). The differences between the earlier time points and the 2014 data were statistically significant (*P* < 0.05). Among those non-clone SA isolates that were PCR positive and whole-genome sequenced, 15 STs were identified (Table S1), five of which were novel sequence types (i.e., they had not been reported previously). Of the 15 STs, 11 were represented by one isolate each, 2 STs were represented by two isolates, 1 ST was represented by five isolates, and 1 ST was represented by three isolates (Table S1).

**TABLE 3:** Occurrence and characteristics of C. jejuni clone SA isolates in dairy cows

Dairy study^*a*^	No. of C. jejuni isolates tested	No. (%) clone SA by:		No. of isolates in which pVir was present	No. of isolates with *tet*(O) located on:	
		PCR	WGS		Chromosome	pTet
2002	205	23 (11.2)	21 (10.2)	2	13	3
2007	627	66 (10.5)	55 (8.8)	1	40	9
2014	576	39 (6.8)	26 (4.5)	2	15	1
Total	1,408	128 (9.1)	102 (7.2)	5	68	13

a NAHMS national surveillance studies.

As carrying tetracycline resistance gene *tet*(O) chromosomally is one of the key features of clone SA isolates from sheep (18), its presence was investigated in the dairy clone SA isolates. Results showed that 81 (79.4%) of the dairy clone SA isolates contained the *tet*(O) gene either in the chromosome (*n* = 68) or on plasmid pTet (*n* = 13), including 13 (61.9%) isolates from the Dairy 2002 study, 51 (92.7%) isolates from the Dairy 2007 study, and 17 (65.4%) isolates from the Dairy 2014 study. In contrast, of the 23 non-clone SA isolates with the whole genome sequenced, 9 isolates harbored a *tet*(O) gene in the pTet plasmid but none of them had *tet*(O) in the chromosome. The pVir plasmid was also found in some of the NAHMS Dairy 2002 (*n* = 2), 2007 (*n* = 1), and 2014 (*n* = 2) study isolates (Table 3).

**FIG 2:**
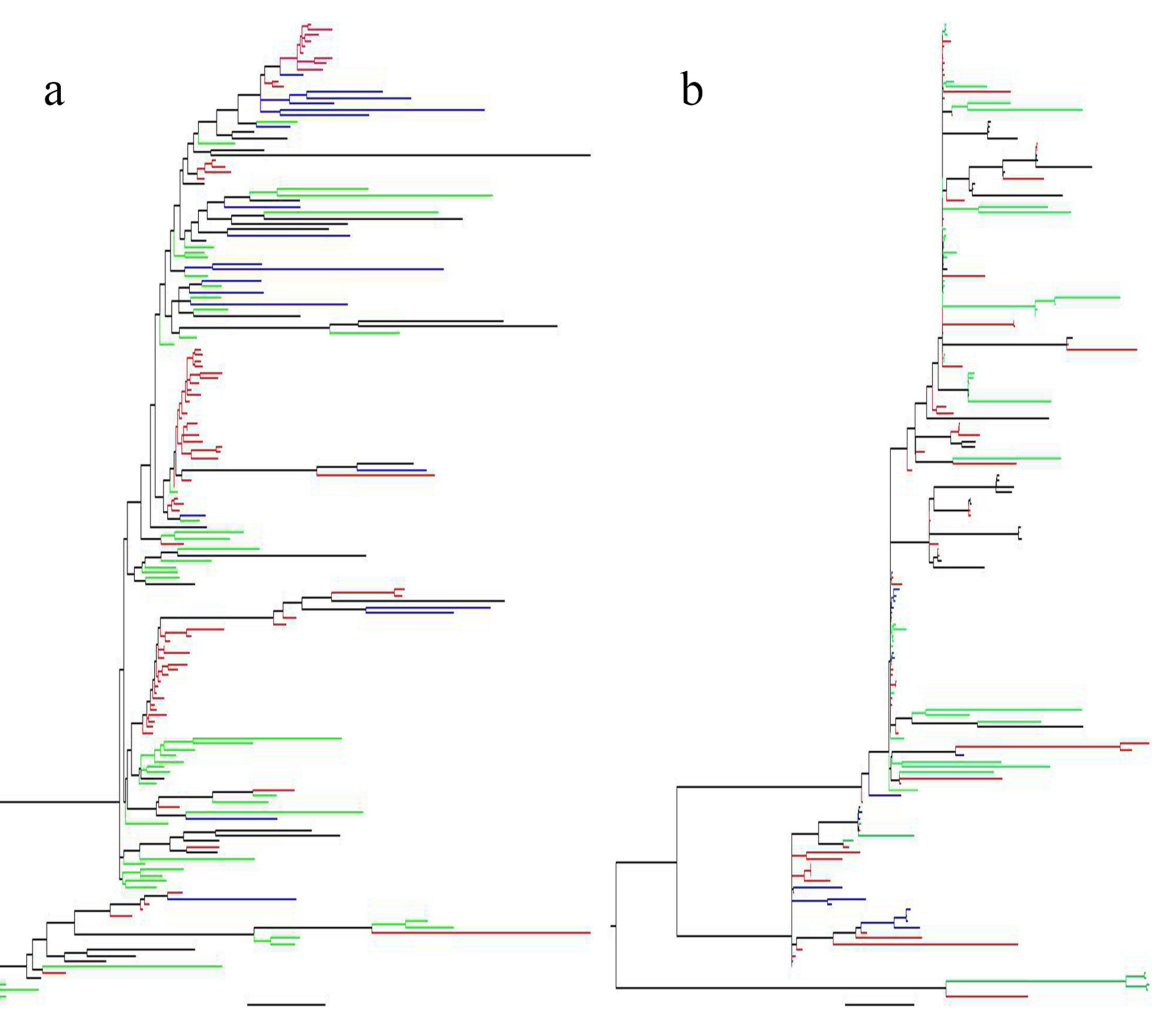
Maximum likelihood phylogenetic tree constructed with the pan-genome (a) and core genome (b) differences among 170 C. jejuni clone SA isolates from sheep and cattle. The clone SA strains are intermixed between sheep and cattle and among the isolation years (2002, 2007, and 2014). The isolates are color-coded based on their source hosts and isolation years: red for sheep, blue for NAHMS Dairy 2002 cattle, green for NAHMS Dairy 2007 cattle, and black for NAHMS Dairy 2014 cattle.

Previously we have determined the whole-genome sequences of clone SA isolates derived from sheep abortion (33). To investigate the genomic relationship between the clinically abortifacient isolates from sheep and the clone SA isolates from dairy cattle feces, maximum likelihood phylogenetic trees were constructed based on the pan-genome (Fig. 2a) and core genome (Fig. 2b) of the clone SA isolates. The trees were constructed with 170 clone SA isolates, including 72 isolates from sheep abortions collected previously (33) and 98 dairy isolates sequenced in this study (the genomic sequences of 3 dairy isolates were excluded due to poor quality). The 72 ovine isolates represented historical and contemporary isolates of clone SA in the United States over the last 2 decades, while the 98 bovine isolates were selected from the NAHMS studies (2002 to 2014). In both trees, clone SA isolates from sheep and cattle were intermixed and formed clusters irrespective of their host species, indicating that clone SA isolates were not host specific. There were some discrete clusters in the trees (Fig. 2). The isolates from the same feedlot and collected in the same year tended to be clustered together, but each of the clusters contained isolates from different feedlots. In addition, we did not observe any specific evolution patterns from the genomic data over the 12-year time span, suggesting that the genome of clone SA was fairly stable.

## DISCUSSION

Results from this study revealed a high prevalence (72.2%) of Campylobacter spp. in feedlot cattle and the distribution of C. jejuni clone SA in both feedlot cattle and dairy cattle in the United States. The identification of C. jejuni as the predominant Campylobacter species in cattle is consistent with previous findings reported by others (17, 26). Considering that genetically diverse C. jejuni strains are present in cattle (32), the prevalence of clone SA (5.8% in feedlot cattle and 7.2% in dairy cattle) is substantial, suggesting that clone SA is well adapted in cattle, similar to the situation in sheep (34). Additionally, we found that European starlings on cattle farms carry C. jejuni, including clone SA, and may serve as a vehicle for the transmission of Campylobacter on farms. Furthermore, WGS analysis of the clone SA isolates collected from dairy cattle during the period from 2002 to 2014 revealed the high genomic stability of the isolates. These findings provide new information on the epidemiology of C. jejuni clone SA in both beef and dairy cattle. Our study documents the distribution of C. jejuni clone SA in beef and dairy cattle, and this work has closed a major knowledge gap regarding the ecology of this zoonotic pathogen in animal reservoirs.

In this study, initial preliminary identification of clone SA was done with a rapid PCR method that targets CJSA_1356, which is one of the variable genes in the capsule locus and is quite specific for clone SA isolates. Previous work has shown the utility of this PCR method for initial screening for clone SA isolates (37). However, this method is not 100% specific for clone SA, which requires further confirmation of the putative clone SA isolates by other methods. For the prospective study on feedlots, we used PFGE and MLST to confirm the identity of clone SA. In the absence of whole-genome sequences, PFGE and MLST are considered the “gold standards” for establishing clonality in Campylobacter isolates (34, 38), and their utility in identifying clone SA was further proven by WGS analysis (33). For the retrospective analysis of the dairy isolates from NAHMS studies, WGS was used to confirm the identity of the clone SA isolates initially identified by PCR. The use of multiple approaches ensured the accuracy of detecting clone SA from a large number of samples.

An interesting finding is that the prevalence rate of clone SA varied significantly in feedlots of different states, highest (7.3%) in Texas and lowest (2.4%) in Colorado. Even within a single state, the prevalence varied from farm to farm. For example, the highest prevalence of clone SA was detected with the no. 4 feedlot in Texas, for which 22 of 47 isolates tested were identified as clone SA, including the clone SA isolate from a starling. The exact reasons for the variable prevalences in different feedlots and states are unknown, but it is possible that the variations are related to differences in management practices that influence transmission and persistence of clone SA in cattle feedlots.

The NAHMS examined Campylobacter prevalence in dairy cows by analyzing individual fecal samples in three separate studies: Dairy 2002 (17), Dairy 2007 (35), and Dairy 2014. By taking advantage of the NAHMS's collections of Campylobacter isolates, we were able to determine the prevalence of clone SA in dairy cattle on a national scale. The availability of isolates from studies conducted in three different years (2002, 2007, and 2014) allowed us to examine the temporal changes in clone SA prevalence over the years. Interestingly, the prevalences of clone SA in 2002 and 2007 were comparable: 10.2% and 8.8%, respectively. However, in 2014, the prevalence decreased to 4.5%, which is significantly different from those in the previous two studies. What is responsible for the decrease of clone SA in dairy cattle is interesting and remains to be determined in future studies.

It was found in this study that 34% of starling fecal samples were Campylobacter positive, with C. jejuni identified as the predominant Campylobacter species. This prevalence rate is within the range of 11.1% to 50.4% previously reported in the United States and outside the United States (36, 39 – 41). PCR screening and molecular typing identified one clone SA isolate in the starling samples. Additionally, PFGE analysis of selected starling isolates revealed genetically diverse strains (Fig. 1), consistent with previous findings for starlings (41, 42). Despite the genetic diversity, two isolates (including a clone SA isolate) showed PFGE patterns indistinguishable from those of the cattle isolates (Fig. 1), suggesting that starlings may play a role in spreading Campylobacter on cattle farms. It should be pointed out that PFGE analysis of the cattle isolates was biased toward putative clone SA isolates and did not represent the entire genetic profiles of the cattle isolates. Thus, the matching between the cattle and starling isolates might be even higher if more cattle isolates (non-clone SA) were analyzed by PFGE. Regardless, results from this study demonstrated frequent isolation of Campylobacter from European starlings on cattle farms and suggest possible two-way transmission of Campylobacter between the two animal species. Interestingly, starling intervention on farms did not affect the overall prevalence of Campylobacter (Table 2), suggesting that starling control alone does not appear to be an effective intervention strategy to reduce cattle fecal shedding of Campylobacter or clone SA in feedlot cattle. This may be due to the fact that multiple interacting factors contribute to the transmission of Campylobacter on cattle farms and control of a single factor has limited impact on its prevalence. Regardless, starlings can be a source for clone SA and can move these isolates between otherwise separate feedlots visited by foraging starlings.

The advances in next-generation sequencing technologies have made it possible to perform high-resolution molecular typing of bacterial isolates. We conducted WGS analysis of the putative clone SA isolates from NAHMS dairy studies, not only for identification of clone SA but also for understanding evolution of clone SA over the 12-year period (2002 to 2014). The WGS analysis confirmed that 102 of the 128 putative clone SA isolates identified by PCR were true clone SA isolates. The genomic data were further used for maximum likelihood phylogenetic tree construction, which revealed that the clone SA isolates derived from 2002 to 2014 are genetically stable and a clear pattern of evolution was not detected as indicted by lack of clustering of the isolates by isolation years (Fig. 2). Inclusion of sheep clone SA isolates (33) in the phylogenetic analysis also revealed that the sheep and cattle isolates are mixed in clustering (Fig. 2), suggesting that the genomic sequences of clone SA isolates are not uniquely associated with host species and the possibility of interspecies (cattle and sheep) transmission of clone SA. These genomic features and the identified wide distribution of clone SA in both beef and dairy cattle suggest that bovine clone SA may serve as an important reservoir for the source of infection in sheep, and clinical abortion induced by C. jejuni clone SA continues to be a significant burden for sheep producers (34).

Tetracycline resistance is an important feature of C. jejuni clone SA isolated from sheep, and acquisition of this resistance trait is like due to antibiotic selection pressure, as tetracyclines are frequently used for control of sheep abortion on farms in the United States (34, 43). The *tet*(O) gene is the only tetracycline resistance determinant identified in Campylobacter so far. Although *tet*(O) is typically carried by plasmids, it is predominantly located in chromosome in clone SA (34). In this study, we found that 79.4% (Table 3) of the dairy clone SA isolates carried the *tet*(O) gene, and in most of the isolates (68/81) it was located on chromosome. However, the *tet*(O) gene in the non-clone SA isolates was all carried by a plasmid. These results are consistent with our previous findings for the sheep Campylobacter isolates (43) and further indicate the advantage of C. jejuni clone SA in dealing with the selection pressure from tetracycline antibiotics. The pVir plasmid was also identified in a small number (5/102) of the clone SA isolates in this study. This plasmid is not required for abortion induction by clone SA (44) and is also infrequently present in sheep clone SA isolates (33). Thus, pVir is not unique to clone SA and its *in vivo* function is still unknown.

In summary, this study revealed detailed molecular and epidemiological features of C. jejuni clone SA in beef and dairy cattle, as well as in European starlings present on cattle farms. These findings underscore the importance of cattle and wild birds in the overall ecology of C. jejuni clone SA in animal reservoirs and provide critically needed information for development of intervention strategies. For example, the high prevalence of C. jejuni clone SA in cattle explains why many of the clone SA-associated foodborne disease outbreaks were attributed to consumption of raw milk (34) and highlights the need to reduce fecal contamination of milk and pasteurize milk before consumption to prevent the transmission of clone SA to humans. Additionally, the variable distribution of clone SA on cattle farms suggests that production practices and/or environmental factors may influence its prevalence and may be managed to control clone SA in cattle. Furthermore, our findings also suggest that control of Campylobacter-induced abortion in small ruminants should consider intervention strategies that mitigate the transmission of clone SA from the cattle reservoir. These findings provide directions for designing future studies to evaluate intervention strategies. Considering the significance of C. jejuni clone SA in ruminant health and food safety, reducing its prevalence on cattle farms will benefit both animal health and public health.

## MATERIALS AND METHODS

### Sample collection and bacterial isolation.

In the prospective BACI study, a total of 3,184 cattle fecal samples were collected from 35 different feedlot herds located in Iowa, Texas, Colorado, Missouri, and Kansas on two different occasions during the period from December 2012 to March 2013. Collection of cattle fecal samples followed methods described previously (45). A sample was collected from a fecal pat only after a cow was observed defecating. Freshly voided fecal pats were scraped with sterile cotton-tipped swabs, and the swabs were immediately placed in 10-ml glass tubes containing Campylobacter thioglycolate broth (CAMPY-THIO). All cattle fecal samples were shipped priority overnight to the testing laboratory. All samples received the next day of collection were accepted and processed to culture Campylobacter as described in a previous study (46). Of note, the fecal samples were collected during a European starling intervention program taking place on the farms (39). The intervention program was designed to examine the role of invasive European starlings in the spread of antibiotic-resistant bacteria in in feedlots. During the intervention, Wildlife Services biologists baited starlings using a 2% solution of DRC-1339 (3-chloro-*p*-toluidine hydrochloride) on treated corn chop. Technical DRC-1339 powder was mixed with water to create a 2% solution. Treated corn chop was soaked in the 2% solution and screen dried. The bait was applied at a concentration of 1:10 treated to untreated corn chop. All DRC-1339 applications were implemented in accordance with label requirements “Compound DRC-1339 Concentrate—Feedlots” (EPA registration 56228-10). In order to determine the effect of this control program on Campylobacter prevalence, approximately one-half of the samples was obtained before the intervention, while the other half was obtained after the intervention.

In addition to cattle fecal specimens, we collected 150 starlings from 7 feedlots (from which postintervention cattle samples were also tested concurrently) within cattle pens and pen lanes during February and March of 2013. All starlings were collected with shotguns, and no birds were collected outside the feedlots. Starling collections followed the methods conforming to agency policy as stated in U.S. Department of Agriculture (USDA), Animal and Plant Health Inspection Service (APHIS), Wildlife Service Directive 2.505 and were approved by the National Wildlife Research Center's (NWRC) Internal Animal Care and Use Committee (NWRC protocol QA-1919). All specimens were individually bagged in sterile Whirl-Paks and stored in coolers until shipping. European starlings were shipped to the NWRC in Fort Collins, CO. All samples received the next day of collection were accepted and processed. All European starling dissections occurred at the NWRC and were conducted using published methods (47). Starling lower gastrointestinal (GI) tracts (duodenum to the cloaca) were removed and placed in sterile Whirl-Paks. To reduce the risk of cross-contamination, we cleaned the starling carcasses, scissors, scalpels, and lab stations with 70% ethanol before the removal of each starling GI tract. Lab mats and gloves were replaced after processing of each starling. The starling GI samples were macerated for 120 s at 200 rpm using a Stomacher 80 paddle blender (Seward Laboratory Systems, Bohemia, NY). Fecal material from the macerated starling GI tracts was squeezed by hand to one corner of the bag and an aliquot was extracted using sterile cotton swabs, making sure to completely saturate the tip of the swab.

In the laboratory, 1 ml of the transport medium containing a fecal swab was added into a tube containing 9 ml of Campylobacter enrichment broth, which was then incubated at 42°C for 48 h under microaerobic conditions (5% O_2_, 10% CO_2_, and 85% N_2_). The enrichment medium was Mueller-Hinton (MH) broth supplemented with Campylobacter-specific selective agents (SR084E and SR117E; Oxoid). From the enrichment culture, an inoculum of 100 μl was streaked onto an MH agar plate containing the same supplements, which was further incubated for 48 h at 42°C under microaerobic conditions. A single Campylobacter-like colony from each sample was subpassaged onto a plain MH agar plate and the pure culture was stored in glycerol stocks at −80°C until further use.

To determine the distribution of C. jejuni clone SA in dairy cattle feces, retrospective collections of Campylobacter isolates from the NAHMS 2002, 2007, and 2014 dairy studies (17, 35, 48) were screened for clone SA. Respectively, 205, 627, and 576 C. jejuni isolates from the three studies were screened for putative clone SA using a specific PCR (see below). Further confirmation of the putative clone SA isolates was performed via whole-genome sequence (WGS) analysis.

### DNA extraction and PCR identification.

DNA was extracted from Campylobacter colonies using single-cell lysis buffer (49) and was used as the template for PCRs. In order to detect and/or differentiate C. jejuni, C. coli, and C. jejuni clone SA, three sets of previously published primers were used. The first primer pair (CCCJ-F, 5′-AAT CTA ATG GCT TAA CCA TTA-3′, and CCCJ-R, 5′-GTA ACT AGT TTA GTA TTC CGG-3′), targeting 16S rRNA, was designed to coidentify C. jejuni and C. coli (50). The second primer pair (mapA-F, 5′-GAG TGC TTG TGC AAC TAA AC-3′, and mapA-R, 5′-ATA GCA TCT TGA GTT GCT CC-3′) was specific for C. jejuni (51). The third PCR primer pair (CJSA_1356F, 5′-TCC CAT TTG GAT GTT GTT GA-3′, and CJSA_1356R, 5′-CAG AAC CTG GCC ACA AAC TT-3′) was used for identification of putative C. jejuni clone SA as described previously (37). C. jejuni IA3902, a clinical isolate of clone SA, was used as a positive control for the PCR, whereas reactions with no DNA template were used as negative controls. Each PCR amplification was carried out in a 25-μl volume containing 16 μl of distilled water, 2.0 μl of template DNA, 10 pmol of each primer, and 5 μl of GoTaq (Promega) green master mix by following the cycling conditions described previously (37, 50, 51).

### PFGE.

Pulsed-field gel electrophoresis (PFGE) analysis of C. jejuni isolates was performed using KpnI by following the PulseNet protocol (Centers for Disease Control and Prevention [CDC]), with minor modifications (29). Briefly, fresh cultures of Campylobacter were embedded in 1% SeaKem Gold agarose (Fisher Scientific, Fair Lawn, NJ) and lysed with proteinase K for 1 h at 55°C in a water bath shaker. The gel plugs were digested with KpnI for 4 h at 37°C. Digested plugs were embedded into 1% agarose and separated by electrophoresis in 0.5× Tris-borate-EDTA (TBE) buffer (Promega) at 14°C for 18 h using a Chef Mapper electrophoresis system (Bio-Rad, Hercules, CA). Gels were stained with ethidium bromide for 30 min and then photographed by using ChemiImager 5500 (Alpha Innotech, CA). The PFGE patterns were analyzed by the GelCompare II v.6.5 software program (Applied Maths, Kortrijik, Belgium) using the Dice similarity coefficient and unweighted-pair group method with arithmetic averages (UPGMA) with 0.5% optimization and 1.5% position tolerance. C. jejuni IA3902 was used as a control for identification of C. jejuni clone SA isolates. A lambda DNA ladder (Bio-Rad) was used as the molecular size marker.

### MLST.

To confirm the PFGE results, multilocus sequence typing (MLST), originally developed by Dingle et al. (52), was performed on 11 representative C. jejuni isolates (10 from cattle and 1 from a starling) from the prospective study on feedlots. Of the 11 isolates chosen, 4 (3 from cattle and 1 from a starling) had PFGE profiles indistinguishable from that of the positive control (C. jejuni IA3902), 4 had minor differences in PFGE patterns, and 3 showed totally different PFGE profiles. The seven housekeeping genes from these 11 C. jejuni isolates were amplified and sequenced using the primer sets described at the C. jejuni MLST website (http://pubmlst.org/campylobacter/), which was developed by Keith Jolley and Man-Suen Chan at the University of Oxford (53). Allele numbers were assigned to the isolates by performing BLAST searches for the assembled sequences using the single-locus query function, whereas sequence types were assigned using the allelic profile query function in the MLST database. Sequences that were identical to existing alleles in the MLST database were assigned the corresponding allele numbers. Novel allele profiles (*n* = 5) were assigned new sequence types (STs) within the MLST database.

### WGS analysis.

The putative clone SA isolates identified by PCR screening from the retrospective NAHMS dairy studies were subjected to WGS. Total DNA was extracted from each isolate using the Wizard Genomic DNA purification kit (Promega) and then used for WGS. The library was constructed using the NEXT Ultra DNA library prep kit (New England BioLabs), and 250-bp paired-end reads were obtained using an Illumina Hiseq2500 (Bionova 42 Biotech Co.). A draft assembly of the sequences of each genome was generated using the *de novo* short-read assembler Velvet (54) and VelvetOptimiser (http://www.vicbioinformatics.com/software.velvetoptimiser.shtml). Draft genome sequences were aligned and the core genome phylogenetic tree was constructed using the single nucleotide polymorphisms (SNPs) by Parsnp in the Harvest package (55), while the pan-genome phylogenetic tree was constructed using binary accessory nucleotide data by Panseq (56). The phylogenetic tree was visualized with FigTree (http://tree.bio.ed.ac.uk/software/figtree). MLST of these isolates was performed using the sequences of the 7 housekeeping genes according to the scheme from PubMLST (https://pubmlst.org/campylobacter/).

### Statistical analysis.

A chi-square test was used to compare the prevalences of Campylobacter before and after starling intervention as well as the prevalences of clone SA in different states for the feedlot cattle and in different years for the dairy cattle. The prevalence of Campylobacter was taken as the response, and the intervention, the state, and the year were taken as the factors. The independence between the factors and the response was tested by SPSS (version 17.0; SPSS Inc., Chicago, IL). *P* values less than 0.05 were considered significant.

## Supplementary Material

Supplemental material
