# Supplementary material for: Wide but Variable Distribution of a Hypervirulent Campylobacter jejuni Clone in Beef and Dairy Cattle in the United States
Source: Appl Environ Microbiol. 2017 Dec 1;83(24):e01425-17. doi: 10.1128/AEM.01425-17 (PMC5717212; doi:10.1128/AEM.01425-17)
Supplement: Supplemental material [file supp_83_24_e01425-17__index.html]

Supplemental material 

# Wide but Variable Distribution of a Hypervirulent Campylobacter jejuni Clone in Beef and Dairy Cattle in the United States

## Supplemental material

- Supplemental file 1 -

  Distribution of sequence types and clonal complexes of selected *C. jejuni* isolates from dairy cattle (Table S1).

  PDF, 580K
