# Supplementary material for: Wide but Variable Distribution of a Hypervirulent Campylobacter jejuni Clone in Beef and Dairy Cattle in the United States
Source: Appl Environ Microbiol. 2017 Dec 1;83(24):e01425-17. doi: 10.1128/AEM.01425-17 (PMC5717212; doi:10.1128/AEM.01425-17)
Supplement: Supplemental material [file AEM.01425-17_zam999118182s1.pdf]

Table. S1. Distribution of sequence types and clonal complexes of selected *C. jejuni* isolates from dairy cattle.

CC	ST	No. of isolates	aspA	glnA	gltA	glyA	pgm	tkt	uncA	Percent (%)
21	8	102	2	1	1	3	2	1	6	88
21	21	1	2	1	1	3	2	1	5	0.8
42	42	1	1	2	3	4	5	9	3	0.8
508	132	1	1	6	22	24	12	28	1	0.8
21	262	5	2	1	1	3	2	1	3	4.0
682	682	1	26	2	9	51	8	46	21	0.8
403	933	1	10	1	59	19	10	5	7	0.8
828	1068	1	33	39	30	78	104	43	17	0.8
61	1244	1	1	1	2	2	225	3	17	0.8
21	2876	3	2	2	1	3	2	1	6	2.4
179	3598	2	1	6	137	176	423	32	3	1.6
685	**8535**	1	1	1	2	4	2	3	6	0.8
21	**8536**	2	40	1	1	3	2	1	6	1.6
21	**8537**	1	2	24	1	3	676	1	6	0.8
NA	**8538**	1	4	6	137	176	40	478	3	0.8
353	**8539**	1	7	17	234	2	10	3	6	0.8

NA: not assigned to any clonal complex; CC: clonal complex; ST: sequence type; bold numbers: new sequence types identified in this study.
